# Background firing rate affects the signal transfer of behavior locked input patterns from Purkinje cells to the cerebellar nuclei

**DOI:** 10.1186/1471-2202-13-S1-P112

**Published:** 2012-07-16

**Authors:** Selva K Maran, Ying Cao, Detlef Heck, Dieter Jaeger

**Affiliations:** 1 Department of Biology, Emory University, Atlanta, Georgia, 30322, USA; 2 Department of Anatomy and Neurobiology, UTHSC, Memphis, Tennessee, 38163, USA

## 

Multielectrode recordings from awake behaving mice revealed rhythmic modulation both in Purkinje cell (PC) simple spike (SS) activity and the cerebellar nuclei (CN) neuron activity that is phase locked to the breathing rhythm. In the present work we were interested in studying how the different levels of background SS activity may affect the transfer of rhythmic SS modulation from PCs to the CN using a multicompartmental CN neuron model.

We created a population of fifty artificial gamma distributed PC spike trains and gave them to the CN neuron model as inputs with 5nS conductance strength [1]. The mean firing rate for a given input was based on the rate histogram distribution constructed from our experimental PC recordings in awake mice, and ranged from 28 to 170 Hz. An identical slow fluctuation in the background firing rate for all 50 inputs was used, reflecting a high correlation of rates observed in vivo as quantified using a Bayesian rate estimation method [2]. The breathing associated rhythmic modulation estimated from a peri-event time histogram of a PC recording was normalized to a % change from mean rate and convolved with baseline firing rate at the timing of recorded breaths.

The synaptic strength of the mossy fiber excitatory inputs was tuned so that the model output gave the best match to both the statistical properties and behavior related modulation of the experimental CN recordings. This match turned out to be excellent, as the mean ISI (recording 29.47, model 29.49), CV (1.072 vs. 0.926), and CV2 (0.74 vs. 0.75) were near identical. The mean breathing related rhythmic modulation in the CN simulation turned out to be higher (60 %) than in the applied PC input trains (12 %). We then separated the breathing modulations obtained in the model output based on whether they occurred at low or high background firing rate. We observed a decrease in modulation depth from 70% to 30% when the baseline firing rate was high in the CN. Similar results were observed in experimental recordings (a decrease from 60% to 30%) which also correlated with an decrease in PC firing rate and a decrease in rhythmicity of breathing during such episodes. To probe the cause of the inverse relationship between CN background rate and breathing modulation depth we used a PC input consisting of a 100 s ramp increase in rate with a fixed breathing modulation depth. This simulation revealed a linear inverse firing rate transformation between PC and CN rates, which also explains the dependence of breathing modulation on background rate. Essentially, since at high rates the absolute breathing related PC rate modulation is higher, this gets translated to greater relative rate modulation of CN neurons at their resulting low mean firing rate. Based on our findings we conclude that the background firing rate of PCs strongly affects the behavioral modulation of the output from the CN. This effect may be important in the cerebellar control or coordination of rhythmic motor behaviors such as breathing, licking, swallowing, and whisking.
